# Blink Perturbation Effects on Saccades Evoked by Microstimulation of the Superior Colliculus

**DOI:** 10.1371/journal.pone.0051843

**Published:** 2012-12-14

**Authors:** Husam A. Katnani, A. J. Van Opstal, Neeraj J. Gandhi

**Affiliations:** 1 Department of Bioengineering, University of Pittsburgh, Pennsylvania, United States of America; 2 Department of Otolaryngology, University of Pittsburgh, Pennsylvania, United States of America; 3 Department of Neuroscience, University of Pittsburgh, Pennsylvania, United States of America; 4 Center for Neural Basis of Cognition University of Pittsburgh, Pennsylvania, United States of America; 5 Department of Biophysics, Donders Institute for Brain, Cognition, and Behaviour, Radboud University Nijmegen, The Netherlands; Charité University Medicine Berlin, Germany

## Abstract

Current knowledge of saccade-blink interactions suggests that blinks have paradoxical effects on saccade generation. Blinks suppress saccade generation by attenuating the oculomotor drive command in structures like the superior colliculus (SC), but they also disinhibit the saccadic system by removing the potent inhibition of pontine omnipause neurons (OPNs). To better characterize these effects, we evoked the trigeminal blink reflex by delivering an air puff to one eye as saccades were evoked by sub-optimal stimulation of the SC. For every stimulation site, the peak and average velocities of stimulation with blink movements (SwBMs) were lower than stimulation-only saccades (SoMs), supporting the notion that the oculomotor drive is weakened in the presence of a blink. In contrast, the duration of the SwBMs was longer, consistent with the hypothesis that the blink-induced inhibition of the OPNs could prolong the window of time available for oculomotor commands to drive an eye movement. The amplitude of the SwBM could also be larger than the SoM amplitude obtained from the same site, particularly for cases in which blink-associated eye movements exhibited the slowest kinematics. The results are interpreted in terms of neural signatures of saccade-blink interactions.

## Introduction

**Figure 1:**
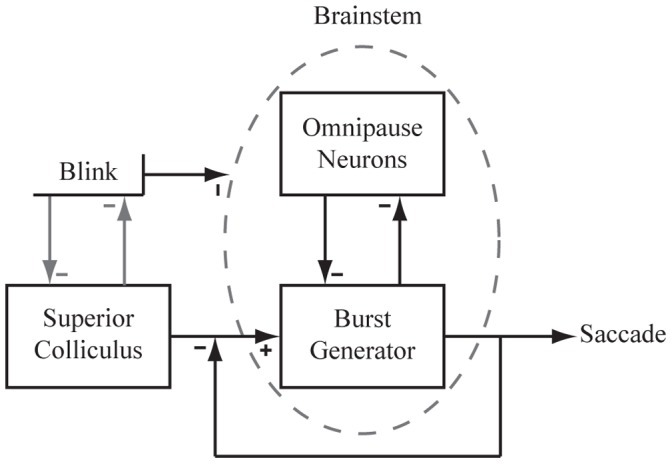
Simplified conceptual scheme of saccade generation. Excitatory projections from the superior colliculus (SC) drive the saccadic burst generator (BG) in the brainstem. Activity from the BG is regulated by a feedback loop to preserve saccade accuracy. Within the brainstem a mutual inhibitory network exists between the omnipause neurons (OPNs) and the BG. Blinks have been shown to affect the brainstem in a manner that suppresses OPN activity. In addition, we incorporate mutual inhibitory effects between the blink and the SC in order to comment on the behavioral correlations seen within our data. Evidence supports the existence of such interaction, although the exact neural correlate is unknown.

**Figure 2:**
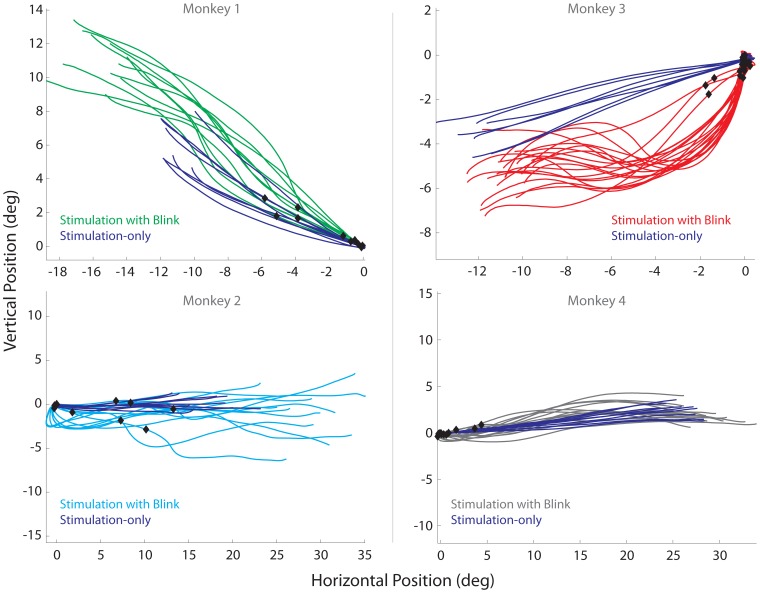
Microstimulation with blink examples. Representative examples of spatial trajectories (horizontal vs. vertical eye positions) shown from the four animals evoked by sub-optimal microstimulation. In all plots, the blue trajectories represent stimulation-evoked reduced amplitude saccades without blinks. Note: mean metrics (horizontal, vertical) evoked by suprathreshold stimulation for monkey 1 (−20.6, 8.5), monkey 2 (29.9, 0), monkey 3 (−23.2, −4.5), monkey 4 (34.8, 5.3). The traces in the other colors represent movements evoked when stimulation was combined with a puff-evoked blink. All traces are offset to the origin with each trace being plotted from stimulation onset to movement offset. Note: Black diamonds superimposed on the trajectories indicate where the blink occurred relative to stimulation onset.

The neural mechanisms underlying the brainstem control of saccades have been well documented (Figure 1; [1], [2]). Briefly, the locus of population activity within the intermediate and deep layers of the superior colliculus (SC) relays a movement command to the burst generator neurons, whose activity patterns are also mediated by a mutually inhibitory functionality with pontine omnipause neurons (OPNs). The OPNs emit spikes at a constant rate during fixation, but the arrival of a saccade command fully quenches activity, effectively disinhibiting the burst generator (BG) and allowing the latter to generate a saccade. The local feedback loop that operates on the BG ensures that the desired saccade command from the SC is produced.

The trigeminal blink reflex is the rapid and transient closure of the eyelids that is invoked most commonly by delivering an air-puff to one eye. It has thus far served as an under-appreciated but useful perturbation tool to test principles of the neural control of saccades. This manipulation has led to three interesting but seemingly incompatible results. One, a blink generated during fixation completely cease the tonic firing rate of OPNs [3]. The temporal features of the OPN pause are better synchronized with the small, loopy blink-related eye movement (BREM) than with the onset and offset of the eyelid movement itself. The result supports the notion that a blink disinhibits the saccadic system. Two, a blink timed to occur during or just prior to a visually-guided saccade attenuates the burst of SC neurons [4]. The interaction of a BREM and saccade alters the spatial trajectory and substantially attenuates the stereotypical bell-shaped velocity profile [5], [6], [7], [8], [9], [10], indicative of a paradoxical, suppressive effect on saccade generation. Three, air-puff pressure that is sufficient to evoke a blink under control conditions fails to trigger a blink when paired with a saccade evoked by supra-threshold stimulation of the SC [11]. This result highlights the inverse effect that the saccadic system can potentially inhibit blink generation.

The objective of the current study was to build on our knowledge of interactions between saccades and blinks. Our approach was to attempt to induce the trigeminal blink reflex during saccades evoked by sub-optimal microstimulation of the SC and to compare the metrics and kinematics of the stimulation-evoked eye movements with and without blinks. We hypothesized that SC inhibition of the blink system will not be potent when using sub-optimal microstimulation and therefore predicted that air-puffs, which are ineffective at producing blinks during supra-threshold SC microstimulation [11], will be consistently successful during sub-optimal stimulation. Our pilot studies confirmed this assertion and hence allowed additional hypotheses to be addressed. As highlighted by the simplified diagram of Figure 1, the complex interplay of excitation and inhibition at various stages of the circuit lead to multiple possibilities. For instance, the population level response generated by sub-optimal stimulation in the SC could be attenuated by the trigeminal blink reflex, which in turn could dampen the dynamics of the stimulation-evoked movement. It is also feasible that a blink occurring with sub-optimal stimulation can prolong the cessation of OPNs, thus extending the temporal window allocated for generating an eye movement. This feature is expected to increase the duration of the stimulation-evoked movement and, depending on the magnitude of attenuation in peak velocity, perhaps also increase the amplitude of the stimulation-evoked movement. Data collected across four animals were consistent with these hypotheses.

## Methods

All procedures were approved by the Institutional Animal Care and Use Committee at the University of Pittsburgh and complied with the guidelines of the Public Health Service policy on Humane Care and Use of Laboratory Animals. Four juvenile, male rhesus monkeys (*Macaca mulatta*) underwent one or more surgeries in a sterile environment and under isoflurane anesthesia. The initial procedure consisted of placing a Teflon-coated stainless steel wire (Baird Industries, Hohokus, NJ) under the conjunctiva of one eye and securing a head-restraint post to the skull. In the second procedure, one cylinder was cemented over a craniotomy. The chamber was placed stereotactically on the skull, slanted posteriorly at an angle of 38° in the sagittal plane. This approach allowed access to both colliculi and permitted electrode penetrations normal to the SC surface. After each surgery, the monkey was returned to its home cage and allowed to fully recover. Post-operatively, antibiotics and analgesics were administered as indicated in the protocol. As a result of the surgically added eye coil and head chamber, all animals were housed individually for safety. The Division of Laboratory Animal Resources continually monitored the animals and provided enrichment in the form of toys, radio, and television. Feeding of nutrition enriched biscuits occurred twice a day as well as the administration of daily fruit, vegetables, and a foraging mix of dried treats. Water was also given to each animal daily.

Behavioral paradigms as well as stimulation and reflex-blink procedures were similar to those described in previous papers [8], [12], [13]. Briefly, all animals were trained to perform the oculomotor gap task. Every trial began with directing the line of sight to a fixation point for 300–500 ms before it was extinguished. Following a 200–400 ms “gap” interval, during which the animal was required to maintain the same eye position, another stimulus was illuminated in the visual periphery. Each animal was permitted 500 ms to redirect its visual axis to the saccade target and hold gaze steady for 300–500 ms to earn a liquid reward. As the animal performed this task, a platinum iridium microelectrode (1.0–1.5 MΩ; MicroProbes for Life Science, Inc., Gaithesburg, MD) was advanced with a hydraulic microdrive (Narashige, Tokyo, Japan). The electrode was driven deeper into the SC until saccadic motor bursts were identified.

The first step of the experiment was to determine the site-specific saccade vector. Microstimulation was delivered during the gap period on a subset of the trials. Constant current stimulation trains were generated using a Grass S88X stimulator in combination with Grass PSIU6 isolation units. Trains consisted of cathodal phase leading, biphasic pulses (0.25 ms). The site-specific vector was determined with high or supra-threshold stimulation conditions (40 µA, 400 Hz, generally 100 ms). If necessary, the depth of the electrode was adjusted to obtain the shortest possible latency of the stimulation evoked saccade (20–40 ms). Next, low stimulation settings were obtained by selecting lower current intensities, frequencies, or both that reliably produced movements (>90% probability of evoking movement). This experimental manipulation also reduced the amplitude of the movements consistently (∼15% or more change in amplitude), as described by previous studies [14], [15], [16], [17]. The sub-optimal stimulation setting could be as low as 10 µA and 100 Hz and differed across sites. Only one set of high and low stimulation-evoked saccades was collected for each data set. In all cases, stimulation duration was manually set (usually 100–300 ms) to ensure that it outlasted the eye movement. Approximately 200 ms after stimulation offset, a target was illuminated at a random location, which the animal had to acquire visually to obtain a reward.

The next phase of the experiment was to investigate the effects of blinks on saccades evoked by sub-optimal stimulation parameters. Four types of gap trials were randomly interleaved to address this goal. *Stimulation-only trials* (20%): As described above, microstimulation was delivered during the gap period and produced stimulation-only evoked movements (SoMs). *Puff-only trials* (10%): A puff of air was delivered to one eye to produce blinks during the gap period. These trials allowed us to characterize the small, loopy eye movement that accompanies the blink, which we refer to as a blink related eye movement (BREM). Eyelid movements were recorded using a small Teflon-coated stainless steel wire that was taped to the eyelid of the eye not implanted with the scleral coil. The eyelid coil signal, described in arbitrary units, was amplified in software to clearly identify eye closure as deflections in the vertical channel. *Stimulation with blink trials* (20%): Microstimulation and air-puff were combined to incorporate the effect of a blink (and prolonged OPN cessation) on a stimulation-evoked saccade, which we refer to as stimulation with blink-evoked movement (SwBM). The air-puff was delivered at random times before and during the stimulation. *Control trials* (50%): These were standard gap trials without stimulation or puff.

Each trial was digitized and stored on the computer’s hard disk for off-line analysis. We used a combination of in-house software and Matlab 7.10.0 (R2011a). Horizontal eye position and vertical eye and eyelid position along with onset and offset times of the stimulation train were stored with a resolution of 1 ms. Component velocities were obtained by differentiating the eye and eyelid position signals. Onset and offset of stimulation-evoked saccades and blink-related eye movements were then detected using a standard 30°/s velocity criteria, respectively.

## Results

Effects of the trigeminal-blink reflex on saccades evoked by supra-threshold and sub-optimal microstimulation parameters were tested on 42 SC sites in four animals (monkey 1: 17, monkey 2: 7, monkey 3: 10, monkey 4: 8). The site-specific vectors, upon rotating into the right hemifield to pool data from left and right SC, spanned approximately 8^o^ to 40^o^ in amplitude and −70^o^ to 40^o^ in direction. When paired with supra-threshold stimulation, air-puffs were completely ineffective in producing blinks for 16 of the 42 sites. For the remaining sites air-puffs yielded blinks on less than 10% of the trials. The result is consistent with previous studies that reported the inability to evoke blinks with supra-threshold collicular stimulation [11], [18]. In contrast, air-puffs delivered during sub-optimal stimulation yielded blinks for over 90% of the trials for every site. The effect was observed for stimulation with low current intensities (14 sites), low pulse-train frequencies (6 sites), or both (22 sites). Given the paucity of combined saccade-blink trials with supra-threshold stimulation, the remaining analyses will focus on sub-optimal stimulation data.

**Figure 3:**
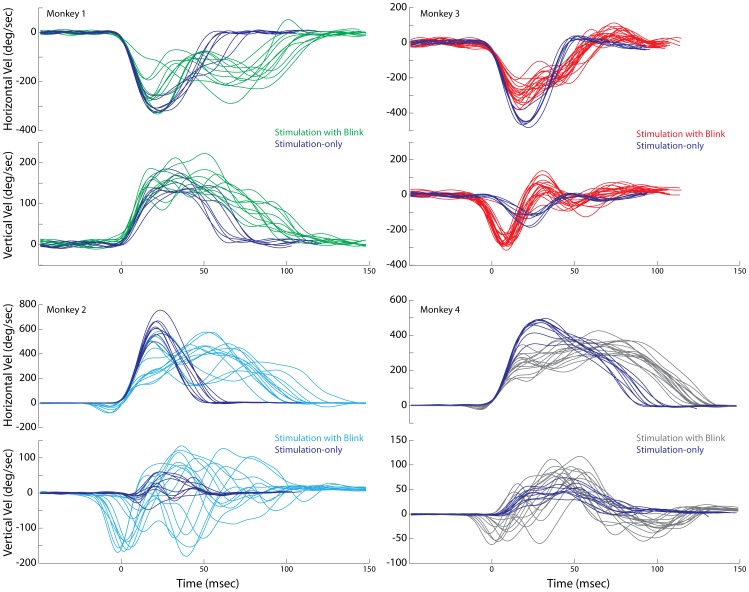
Temporal waveforms. An alternate representation of the data illustrated in Figure 2. Horizontal and vertical eye velocity is plotted as a function of time for stimulation-evoked saccades with and without blink perturbations. All traces are aligned on saccade onset. All other configurations are the same as in Figure 2.

**Figure 4:**
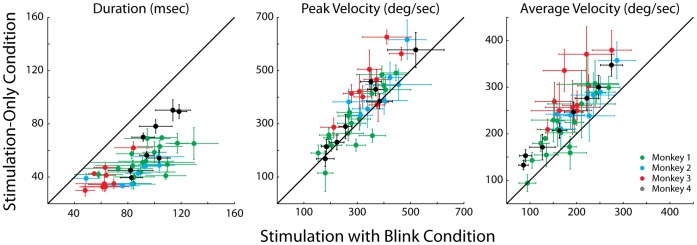
Kinematics. The scatter plots compare the duration (left), peak velocity (middle) and average velocity (right) of saccades evoked from stimulation-only trials and stimulation-with-blink trials. Each dot represents the mean value from one stimulation site, and the error bars represent one standard deviation. The four colors correspond to the four animals, as indicated in the key.

Figure 2 shows representative examples of spatial trajectories of stimulation-only evoked movements (SoMs, blue trajectories) and stimulation with blink evoked movements (SwBMs, green, cyan, red, and gray trajectories) observed at four sites, one from each animal. All traces are aligned and shifted to start at stimulation onset and at the origin, respectively. The black diamonds superimposed on the SwBMs trajectories indicate the eye position at the time of blink onset. The representative examples demonstrate that the relatively straight spatial trajectories of SoMs can become markedly curved during SwBMs. Figure 3 plots the same data as temporal profiles of horizontal and vertical velocities aligned on saccade onset. It is clear that the durations of SwBMs are longer than of SoMs evoked from the same site and with identical stimulation parameters. Furthermore, the blink produced a pronounced attenuation in the stereotypical bell-shaped velocity profile associated with saccades. Figure 4 compares the average duration, peak velocity, and average velocity (saccade amplitude divided by its duration) between SoM and SwBM conditions for each stimulation site from the four animals. The increase in duration and decrease in average velocity was statistically significant for all four animals (signtest, p<0.001), while the comparison of peak velocity showed no statistical difference for any monkey (signtest, p>0.05). A weaker effect on peak velocity is not unexpected based on the velocity waveforms shown in Figure 3. In many cases, the initially dampened eye movement is often followed by a reacceleration in mid-flight. The peak velocity of the acceleration component is often comparable to that of the average SoM but occurs much later in the movement; in some cases, the peak velocity of re-accelerated SwBM movement was even higher, as indicated by the three green points below the unity line. Since the summary analysis extracted the peak velocity (Figure 4) across the entire duration of the movement, the magnitude of the reaccelerated component contributes negatively to the statistical evaluation. The average velocity measure, in contrast, circumvents this confound and more aptly conveys the attenuation observed with SwBMs.

**Figure 5:**
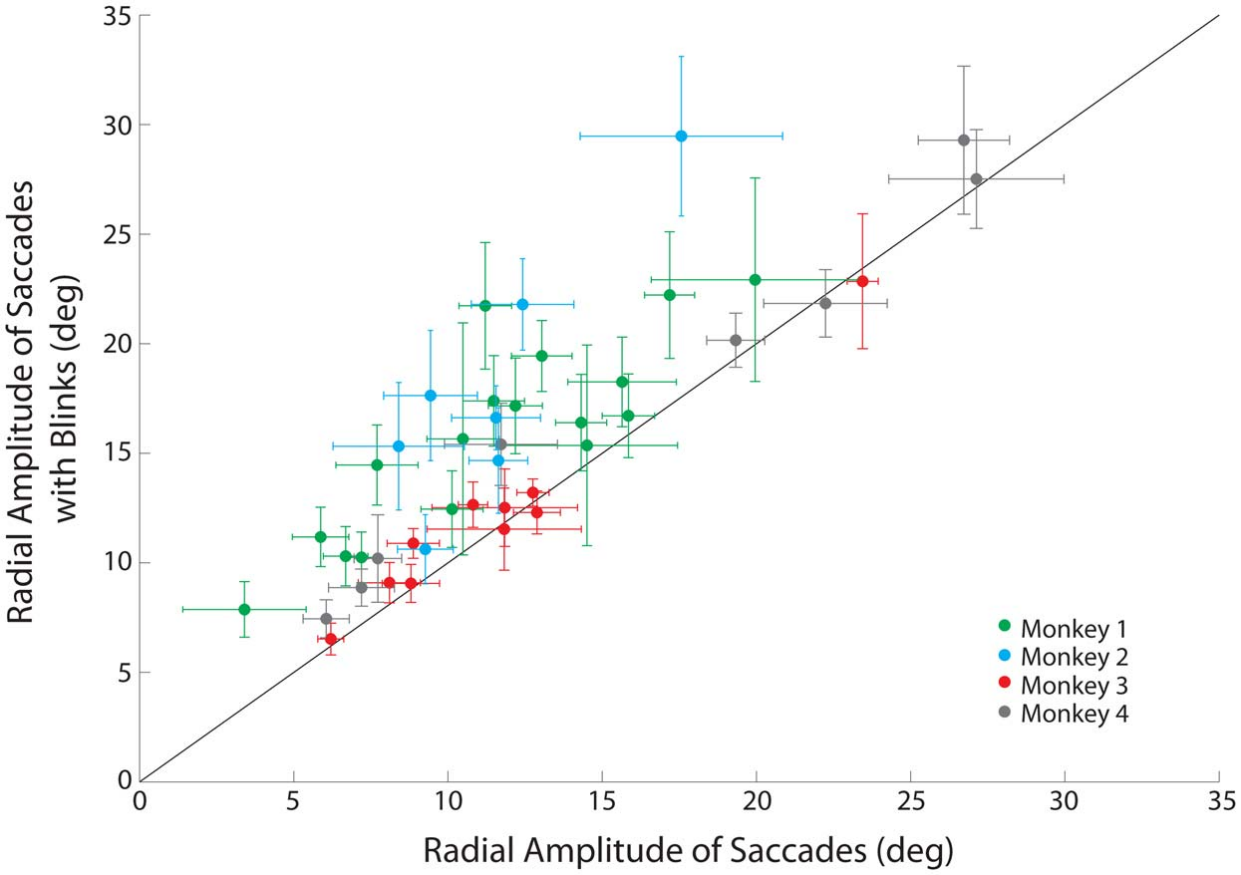
Comparison of radial amplitude. Comparison of mean radial amplitudes for stimulation-evoked saccades without and with a puff-evoked blink. Green dots represent values from monkey 1; cyan, monkey 2; red, monkey 3; gray, monkey 4. Error bars represent one standard deviation from the mean; solid line marks unity slope. The majority of stimulation sites lie above the unity line, indicating an increase of saccade amplitude due to the blink-saccade interaction.

Another result that can be extracted from the spatial trajectories plots (Figure 2) is that the radial amplitude of SwBMs can be larger than the SoMs. This is particularly appreciable for the two sites illustrated in the left column. Figure 5 compares the mean radial amplitudes of SoMs and SwBMs on a site-by-site basis for the four animals. The radial amplitude was significantly larger for SwBMs across the entire dataset as well as for monkeys 1 and 2 (green and cyan dots; paired signtest, p<0.001), but not for monkeys 3 and 4 (red and gray dots; paired signtest, p>0.05).

We wondered whether the significant hypermetria observed for monkeys 1 and 2 could have been simply due to a linear superposition of the BREM contribution. To test for this, we subtracted the maximum horizontal and vertical excursions of BREMs collected during puff-only trials (see Methods) from the endpoints of SwBMs. While this step naturally reduces the amplitudes of the SwBMs and shifts them closer to the amplitudes of the SoMs (equivalently a downward shift closer to the line of unity in Figure 5, data not shown), the hypermetria in monkeys 1 and 2 remained statistically significant (signtest, p<0.001). Therefore, the large radial amplitudes of SwBMs for monkeys 1 and 2 were not merely the result of an added BREM contribution.

**Figure 6:**
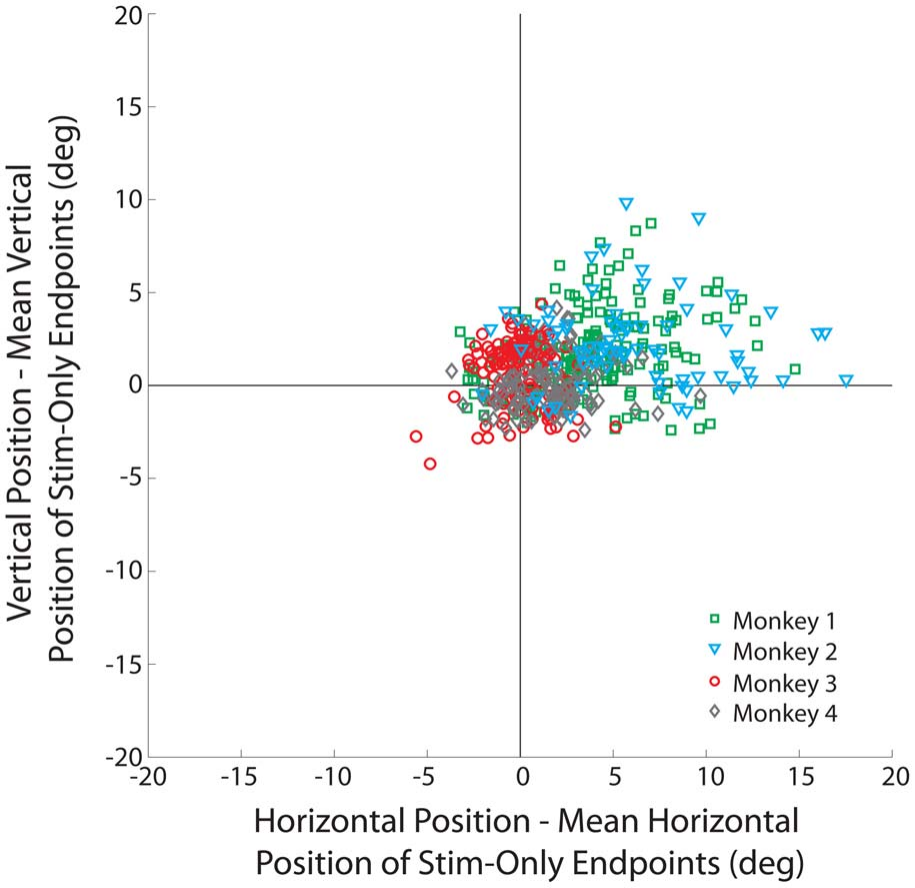
Distribution of dysmetria induced by the blink perturbation. Each point represents the horizontal and vertical endpoint position of a stimulation-with-blink movement after subtraction of the endpoint of the mean stimulation-only movement obtained from the same stimulation site. Each dot represents one trial, and data from all trials across all stimulation sites are included in the plot. Green squares represent data for monkey 1; cyan triangles, monkey 2; red circles, monkey 3; gray diamonds, monkey 4. Note that the absolute values of endpoints were used to standardize alignment for movements in opposing directions.

We also tested whether the change in amplitude was in the direction of the stimulation-evoked vector, as opposed to a direction merely mediated by the BREM. We note that we used the absolute values of endpoints in this analysis in order to standardize alignment for movements in opposing directions. We subtracted the mean endpoint of the SoMs from the endpoint of each SwBM endpoint for each data set. As a result, the SwBM endpoints were plotted relative to the mean endpoint of SoMs for the corresponding stimulation site (Figure 6; monkeys 1, 2, 3, and 4: green squares, cyan triangles, red circles, and gray diamonds, respectively). For monkeys 1 and 2, the mean horizontal and vertical components of SwBM endpoints were significantly shifted away from zero (t-test: p<0.001) and into the upper-right quadrant, verifying that the overshoot occurred in the direction of the stimulation-evoked saccade (this can also be appreciated from the examples in Figure 2, left column). In contrast, monkey 3 (red) exhibited a significant overshoot in the vertical dimension, while monkey 4 (gray) produced a significant overshoot in the horizontal component only (t-test: p<0.001). Both findings can be appreciated by the spatial trajectories shown in Figure 2, right column; nevertheless, the number of points showing small vertical overshoot in monkey 3 and small horizontal overshoot in monkey 4, did not significantly contribute to the overall change in amplitude seen across all sites for each monkey (Figure 5). Moreover, we obtained no significant overshoot in monkeys 3 and 4 (t-test: p>0.05) when subtracting the maximal horizontal and vertical excursions of BREMs collected during puff-only trials from the endpoints of SwBMs. Thus, the observed component overshoots seen in these two animals could have potentially corresponded with BREM contributions seen in SwBMs.

**Figure 7:**
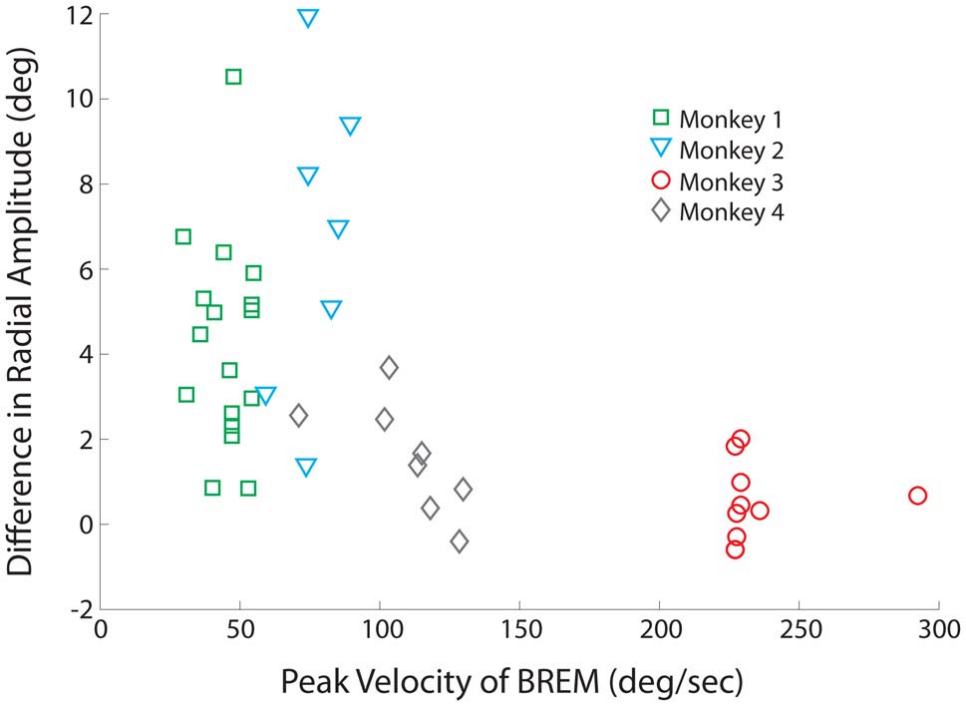
Correlation with BREM kinematics. The peak velocity of BREM movements versus the change in mean radial amplitude of stimulation-evoked saccades colliding with a puff-evoked blink. Green squares correspond to monkey 1; cyan triangles, monkey 2; red circles, monkey 3; gray diamonds, monkey 4.

To further probe the individual differences between animals, we wondered whether the presence or absence of hypermetria could be correlated to other factors. We reasoned that, even if the OPNs remain quiescent because of the blink, an absent or substantially weakened premotor drive to the burst generator would end the movement and prevent overshoot. If so, why would the saccadic motor command be (more) attenuated in monkeys 3 & 4 compared to the other two animals? Previous work [4] has shown that a blink evoked during a saccade suppresses the burst of premotor neurons in the SC. There is a modest initial suppression linked to the time of puff, which is likely mediated through the trigeminocollicular projection [19], but the pronounced attenuation is observed when the saccadic eye movement overlaps with the BREM. Furthermore, we expect that this relationship applies also for saccades evoked by suboptimal stimulation because, as we have argued recently [17], the population SC output is most likely not entrained to the stimulation train. Instead, it likely reflects a network level response that is comparable for stimulation-evoked and target-activated responses. Accordingly, we hypothesized that the faster the BREM kinematics, the stronger the attenuation in the SC burst or, equivalently, the weaker the saccade motor command and the smaller the saccade overshoot. We therefore compared the peak velocity of BREMs as a function of the change in radial amplitude (SwBM-SoM). Since BREM signals cannot be readily extracted from SwBMs, we used the peak velocity of the average BREM trace from puff-only trials (the eyelid profiles were similar for blinks evoked on puff-only and SwBM trials; data not shown) and plotted this against the average difference in radial amplitude for each stimulation site (Figure 7). The illustration reveals that there is little variability in BREM peak velocity within an animal, precluding a meaningful within-animal analysis. However, BREM kinematics did vary substantially across animals [20], which can be appreciated by the different colored symbols in Figure 7.When the data are pooled across animals the overall correlation demonstrates an inverse relationship (correlation coefficient = −0.6, p<0.001), in which higher BREM peak velocities significantly correlated with smaller differences between SoM and SwBM amplitude. Therefore, the size of the blink perturbation seems to indicate a potential level of impedance on induced activity, and could explain the variability of evoked amplitude increases across monkeys.

In an attempt to identify additional trends that could account for the distribution of radial amplitude differences between SoMs and SwBMs, within an animal and across animals, we also correlated the differences with numerous saccade features (i.e., peak velocity of movements, BREM onset and offset relative to stimulation onset and offset, location on the SC motor map, BREM duration, eye lid peak velocity, and latency differences), but found no statistically significant trends.

## Discussion

We have shown that, when paired with SC stimulation, the trigeminal blink reflex can be triggered reliably only when using sub-optimal stimulation parameters. The combined saccade-blink movement displayed a deviation in the spatial trajectory, attenuation of the eye velocity profile, and an increase in movement duration. In two of the four animals, the amplitude of the stimulation-evoked movement was consistently larger in the presence of a blink; the increase in amplitude was not due to an additive effect of the BREM. This spectrum of effects can be accounted for by complex combination of excitatory and inhibitory interactions at various nodes of the oculomotor circuit (Figure 1).

### Vigor of SC Output Mediates Blink Occurrence

Previous studies have demonstrated that the trigeminal blink reflex is rarely evoked during supra-threshold stimulation of the SC [11], [18]. While we confirmed this finding, we also found that a blink can be evoked readily when paired with weaker stimulation parameters. This result collectively suggests that the vigor of SC output can control the likelihood of producing a blink. In accordance with this notion, naturally occurring blinks are less likely to accompany small- and medium- amplitude saccades [20], [21], which are driven by high discharge rates in the rostral and middle SC, compared to larger amplitude movements driven by lower discharge rates from caudal regions [22], [23], [24]. Furthermore, when blinks do accompany small-amplitude movements, such as with memory-guided saccades, the magnitude of eyelid depression is also small (A. S. Powers, personal communication). Thus, there appears to be a direct correlation between the level of SC activity, whether generated in response to a stimulus or stimulation, and the probability of inhibiting a blink. The neural pathway critical in inhibiting the blink reflex is not clear, but it is not likely to be mediated by the putatively excitatory projection from the deep SC layers to the facial nucleus region containing motoneurons that innervate the orbicularis oculi muscles [25].

### Suppressive Effects of Blinks on Saccade Generation

Previous studies based on visually-guided saccades have provided evidence that the blink reflex and the eye movement associated with it impose a suppressive effect on the saccadic system. Behaviorally, this is evident from the attenuation in the kinematics and temporal features of eye velocity waveforms (Figures 2–4; [6], [8], [9]). At the neural level, ∼10 ms after the onset of the air-puff, a subset of SC neurons displays a transient suppression that is likely mediated through the trigemino-collicular pathway [4], [26]. The high frequency burst of SC neurons is also grossly modulated during the ensuing blink-perturbed saccade. Interestingly, the suppression of the SC burst is not nearly as robust when a non-reflexive, gaze-evoked blink is endogenously generated with the saccade [4], for which the perturbation in eye velocity was not as robust as during the puff-triggered blink. These results collectively suggest that the rigor of SC burst is modulated not only by the air-puff but also by the temporal features of the combined saccade-blink movement. This reasoning leads us to propose that the more pronounced the effect of the blink perturbation on the saccade trajectory, the stronger the attenuation of SC activity.

The results of our current study suggest that the suppressive effect of blinks on the saccadic system also applies to movements evoked by sub-optimal stimulation of the SC. We have argued recently [17] that during microstimulation, network properties within the SC dominate the stimulation-induced pulse train and produce a population level response that largely resembles that associated with target-directed saccades. Furthermore, the vigor of activity changes with stimulation parameters, much like the population activity is modulated by the presence or absence of a visual target [27]. Accordingly, saccades evoked by supra-threshold stimulation obey main-sequence properties, whereas movements evoked by sub-optimal parameters exhibit lower peak velocities [14], [15], [16], [17]. Our insight, however, does not discount the possibility that a subset of neurons within the population do emit a spike for each pulse delivered through the electrode and that the entrainment could become the dominant component for high stimulation parameters, such as when stimulation evokes stair-case saccades with a constant velocity movement during the inter-saccadic intervals [28], [29]. We hypothesize that the effect of a reflexive blink on the population response is comparable for stimulation-evoked and visually-guided movements. Hence, a blink evoked during a stimulation-evoked saccade suppresses the population response and reduces peak and average velocities and, furthermore, the attenuation in neural activity scales with BREM kinematics.

### Disinhibitory Effects of Blinks on the Saccadic System

Omnipause neurons (OPNs) located along the midline in the oculomotor paramedian pontine reticular formation are traditionally considered to gate saccadic eye movements [30]. Interestingly, the tonic activity of OPNs also ceases abruptly during blinks induced during fixation, and the temporal aspects of the OPN pause are better associated with the loopy BREM than with the eyelid closure itself [3]. Intracellular and local field potentials recorded during head-restrained saccades reveal a signal that resembles the reciprocal of the eye-velocity waveform [31], [32]. Hence, it seems reasonable to assume that the OPN membrane potential is the inverse of the BREM velocity profile during OPN inhibition associated with a blink produced during fixation.

Reflexive blinks evoked during visually-guided saccades can exhibit pronounced attenuation in the velocity waveforms [6], [8], [9], which is associated with marked reduction of the SC burst [4]. If OPN inhibition were mediated solely by the eye velocity command resulting from the SC output, then a significant reduction in eye velocity could lead to a premature resumption of activity in the OPNs, which would arrest the movement in mid-flight and well short of the desired endpoint. Contrary to this prediction, behavioral studies have demonstrated instead that blink-perturbed saccades are equally as accurate as control saccades [6], [8], [9]. Thus, the OPNs must remain inhibited for the duration of the perturbed movement, and we hypothesize that the blink and/or BREM related signals fulfill this function by supplementing the inhibition imposed by the BG (Figure 1). The OPNs resume when the local feedback loop drives the motor error to zero, thus preserving the accuracy of the movement. Furthermore, it should be realized that in order for the local feedback loop to compensate for the blink-induced perturbation, both the interval of pause in OPNs and movement duration must be prolonged, which is indeed the case [6], [8], [9].

Now we consider how this idea extrapolates to blinks combined with saccades evoked by SC microstimulation. It is known that the tonic activity of OPNs ceases during saccades evoked by SC stimulation [33] and, presumably, the intracellular membrane potential reflects the reciprocal of eye velocity as it does during visually-guided saccades. While supra-threshold SC stimulation evokes a site-specific vector [34], [35], [36], sub-optimal stimulation evokes smaller amplitude saccades with slower velocity waveforms [14], [15], [16], [17]. We hypothesize that a weaker SC output associated with sub-optimal stimulation (see above; [17]) yields a weakened oculomotor drive to the brainstem BG and therefore moderate hyper-polarization of the OPNs. Note that even though the membrane potential is predicted to be weakly hyperpolarized during slow and sluggish saccades, the OPNs do remain completely inhibited as gauged from the absence of spikes. When the velocity drops below some threshold, the OPNs resume their discharge, inhibit the burst generator, and stop the saccade short of its intended endpoint before the local feedback is able to drive the motor error to zero (see Figure 1). We reasoned that inducing a blink during the stimulation-evoked saccade would extend the period of OPN inhibition and grant the BG a larger temporal window to integrate the SC output into a movement. This would result in an increase in saccade duration, which was strongly supported by the data across all four animals (Figure 4).

The results of Figure 5 demonstrate that saccade amplitude increased consistently with the blink perturbation for two animals, while there was no change in the other two. As it seems logical to assume that the OPNs pause for the entire, prolonged duration of the combined blink-saccade movement, some other explanation must account for the differences in saccade metrics across animals. As discussed above, the population SC response, and therefore the oculomotor drive, is transiently attenuated by the blink perturbation, whereby the magnitude of attenuation increases with the strength of BREM kinematics. This suppressive effect is countered by a stimulation-entrained activity in a subset of neurons. We propose that if the blink-induced suppression in the population SC response is modest, which we associate with slow BREM kinematics (monkeys 1 & 2; Figure 7), then the additional entrained spikes augment the oculomotor drive and increase desired saccade amplitude. On the other hand, when the BREM kinematics are faster (monkey 3 & 4; Figure 7), which would impose a stronger suppression on the population SC activity, the stimulation-entrained activity is not sufficient to boost the oculomotor drive. In this case, the desired saccade amplitude remains unaffected, despite the blink perturbation.

### Significance to Motor Decoding in the Oculomotor System

Saccade generation requires the brainstem BG to decode population activity emanating from various oculomotor structures, including the SC. The vector summation with saturation (VSS) model proposes that the cumulative sum of the collicular output drives the BG until saturation constrains the sum and terminates the movement [23], [37], [38]. We recently concluded that the saturation function could be implemented in multiple ways, including intracollicular interactions and gating by the OPNs [13]. The blink manipulation in the present study explored this latter potential mechanism. Presumably, the blink prolongs the duration of OPN suppression, which would effectively allow the cumulative summation of SC activity to occur over a longer duration and hence generate a larger movement. Consistent with this prediction, we did observe larger amplitude movements in two animals when a blink coincided with stimulation, although the suppressive effects of blinks on the saccadic SC drive may in turn have reduced the increase [4], or even abolished it entirely in the other two animals. Clearly, the result would have been even more compelling if the evoked vector amplitude, in the presence of a blink, exceeded the site-specific saccade vector. Unfortunately, the inability to evoke blinks during supra-threshold stimulation, and suppressive effects of the blink on the saccadic drive, may have prevented this assessment.
